# *catena*-Poly[[[tetra­kis­(4-methyl­pyridine-κ*N*)copper(II)]-μ-sulfato-κ^2^ *O*:*O*′] 4.393-hydrate]

**DOI:** 10.1107/S1600536811006325

**Published:** 2011-02-26

**Authors:** Naveed Alam, Muhammad Shahid, Muhammad Mazhar, Saad Al-Jassabi, Matthias Zeller, Allen D. Hunter

**Affiliations:** a Department of Chemistry, Quaid-I-Azam University, Islamabad 45320, Pakistan; b Department of Chemistry, Faculty of Science, University of Malaya, Lembah Pantai, 50603 Kuala Lumpur, Malaysia; c Department of Biochemistry, Faculty of Science, University of Malaya, Lembah Pantai, 50603 Kuala Lumpur, Malaysia; d STaRBURSTT-Cyberdiffraction Consortium at YSU and Department of Chemistry, Youngstown State University, 1 University Plaza, Youngstown, Ohio 44555, USA

## Abstract

The structure of the title compound, {[Cu(SO_4_)(C_6_H_7_N)_4_]·4.393H_2_O}_*n*_, consists of Cu^2+^ ions surrounded in a square-planar fashion by 4-methyl­pyridine ligands, forming two crystallographically independent Cu{H_3_C(C_5_H_4_N)}_4_ units that are both located on crystallographic inversion centers. The Cu(4-methyl­pyridine)_4_ units are, in turn, connected with each other *via* bridging sulfate anions, leading to the formation of infinite [Cu{H_3_C(C_5_H_4_N)}_4_SO_4_]_*n*_ zigzag chains along [001]. The completed coordination spheres of the Cu^2+^ ions are slightly distorted octa­hedral. The axial Cu—O bonds are elongated [average length = 2.42 (4) Å] compared to the equatorial Cu—N bonds [average length = 2.043 (2) Å]. The inter­stitial space between the chains is filled with uncoordinated water mol­ecules that consolidate the structure through O—H⋯O hydrogen bonding. One of the five crystallographically independent solvent water mol­ecules is partially occupied with an occupancy factor of 0.396 (4). Due to hydrogen bonding between symmetry-equivalent water mol­ecules across inversion centers, several of the water H atoms are disordered in 1:1 ratios over mutually exclusive positions. The crystal under investigation was found to be non-merohedrally twinned in a 0.789 (1):0.211 (1) ratio by a 180° rotation around the reciprocal *b* axis.

## Related literature

For the structures of related binuclear copper(II) complexes, see: Shahid *et al.* (2008 ▶, 2009 ▶). 
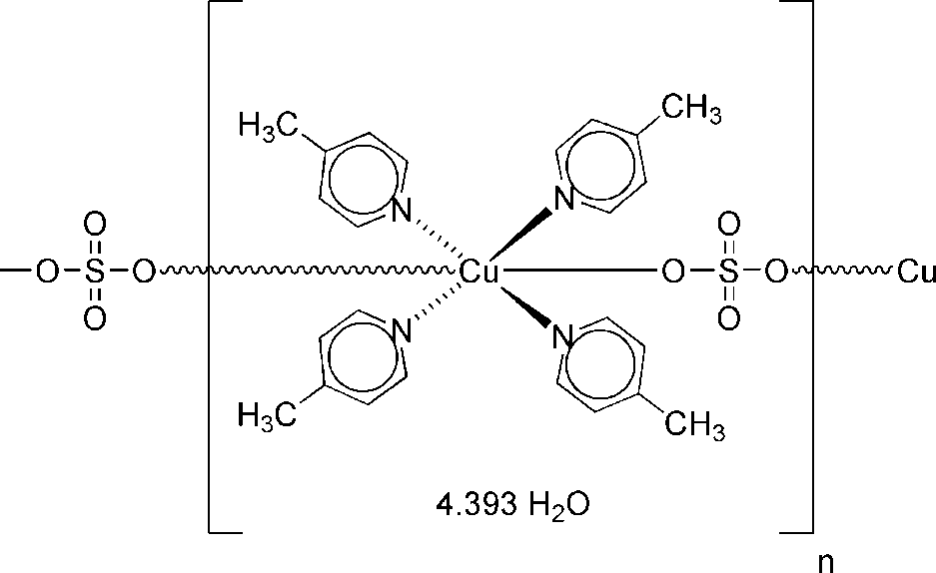


## Experimental

### 

#### Crystal data

[Cu(SO_4_)(C_6_H_7_N)_4_]·4.393H_2_O
*M* *_r_* = 611.27
Triclinic, 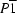

*a* = 10.4688 (12) Å
*b* = 11.6327 (14) Å
*c* = 12.8300 (15) Å
α = 78.672 (3)°
β = 87.609 (3)°
γ = 67.571 (3)°
*V* = 1415.2 (3) Å^3^
*Z* = 2
Mo *K*α radiation
μ = 0.90 mm^−1^
*T* = 100 K
0.60 × 0.45 × 0.40 mm

#### Data collection

Bruker SMART APEX CCD diffractometer
Absorption correction: multi-scan (*TWINABS*; Bruker, 2008 ▶) *T*_min_ = 0.607, *T*_max_ = 0.746
16814 measured reflections
6963 independent reflections
6170 reflections with *I* > 2σ(*I*)
*R*_int_ = 0.033

#### Refinement

*R*[*F* ^2^ > 2σ(*F* ^2^)] = 0.046
*wR*(*F* ^2^) = 0.118
*S* = 1.06
6963 reflections
361 parameters
H-atom parameters constrained
Δρ_max_ = 0.68 e Å^−3^
Δρ_min_ = −0.61 e Å^−3^

### 

Data collection: *SMART* (Bruker, 2002 ▶); cell refinement: *SAINT* (Bruker, 2009 ▶); data reduction: *CELL NOW* (Bruker, 2009 ▶) and *SAINT*; program(s) used to solve structure: *SHELXTL* (Sheldrick, 2008 ▶); program(s) used to refine structure: *SHELXTL*; molecular graphics: *SHELXTL* and *Mercury* (Macrae *et al.*, 2008 ▶); software used to prepare material for publication: *SHELXTL*.

## Supplementary Material

Crystal structure: contains datablocks I, global. DOI: 10.1107/S1600536811006325/wm2458sup1.cif

Structure factors: contains datablocks I. DOI: 10.1107/S1600536811006325/wm2458Isup2.hkl

Additional supplementary materials:  crystallographic information; 3D view; checkCIF report

## Figures and Tables

**Table 1:** Hydrogen-bond geometry (Å, °)

*D*—H⋯*A*	*D*—H	H⋯*A*	*D*⋯*A*	*D*—H⋯*A*
O5—H5*A*⋯O6^i^	0.85	2.04	2.887 (4)	173
O5—H5*B*⋯O4^ii^	0.85	2.01	2.843 (4)	168
O6—H6*A*⋯O6^i^	0.84	2.34	2.983 (6)	133
O6—H6*B*⋯O4^ii^	0.85	1.92	2.762 (4)	172
O6—H6*C*⋯O8	0.85	1.98	2.804 (5)	163
O7—H7*A*⋯O5	0.84	2.16	2.906 (4)	148
O7—H7*B*⋯O3^iii^	0.85	2.13	2.923 (4)	157
O8—H8*A*⋯O3^ii^	0.84	2.42	2.788 (4)	107
O8—H8*B*⋯O6	0.85	2.04	2.804 (5)	149
O8—H8*C*⋯O8^iv^	0.85	1.87	2.710 (7)	176
O9—H9*A*⋯O6	0.84	2.48	3.210 (7)	145
O9—H9*B*⋯O3^ii^	0.84	2.22	3.063 (7)	175
O9—H9*B*⋯O4^ii^	0.84	2.71	3.275 (7)	126

Symmetry codes: (i) 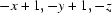; (ii) 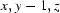; (iii) 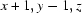; (iv) 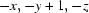.

**Fig. 1.**
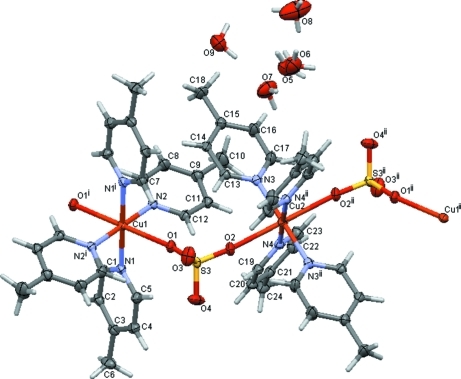
View of the molecular structure of the title compound with displacement ellipsoids drawn at the 50% probability level with atom labeling. Symmetry transformations used to generate equivalent atoms: (i) -x + 1,-y + 2,-z + 1; (ii) -x + 1,-y + 2,-z. For the water molecules all disordered H atoms are shown.

**Fig. 2.**
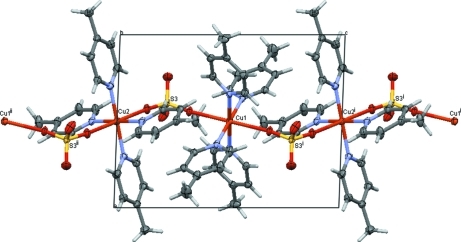
View of the polymeric zigzag chains along the [001] direction. Symmetry transformations used to generate equivalent atoms: (i) -x + 1, -y + 2,-z + 1; (ii) -x + 1,-y + 2,-z.

**Fig. 3.**
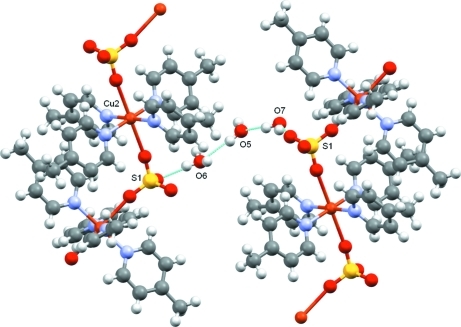
Interdigitation between parallel zigzag chains and hydrogen bonding. View is down the c axis along the direction of the polymeric chains shown in Fig. 2. Note the interdigitation of tolyl groups of parallel chains along the a axis and the water filled areas between chains along the b axis. Hydrogen bonds are indicated by dashed light blue lines. Bands of H–bonded water molecules stretch infinitely parallel to the a axis.

**Fig. 4.**
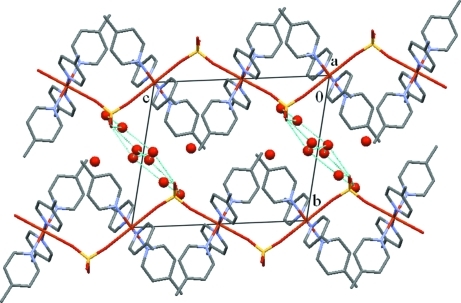
Packing and hydrogen bonding in the structure of the title compound. View is down the a axis. Hydrogen bonds are indicated by dashed light blue lines (H atoms omitted for clarity). Layers of H bonded water molecules stretch infinitely parallel to the a-axis. The single water molecule O9 that is not part of the water cluster is clearly visible in this view.

## supplementary crystallographic information

### Comment

In relation to our previous work on the structural chemistry of copper complexes (Shahid *et al.*, 2008; 2009) the title compound was prepared as the unintended product of the reaction of CuSO_4_^.^5H_2_O with pyrolidine in acetone and 4-methylpyridine.

In the title structure (Fig. 1), two crystallographically independent Cu^2+^ ions are each located on an inversion center. Their coordination environments are distorted octahedral, with a CuN_4_O_2_ set of ligating atoms, composed of four N atoms from four 4-methylpyridine groups and two O atoms of two sulfate anions. The equatorial plane is made up of four N atoms of the 4-methylpyridine ligands, N1, N1^i^, N2 and N2^i^ for Cu1 and N3, N3^ii^, N4 and N4^ii^ for Cu2 (symmetry operators (i) -*x* + 1, -*y* + 2, -*z* + 1; (ii) -*x* + 1, -*y* + 2, -*z*.), with distances ranging between 2.041 (2) and 2.046 (2) Å. The O atoms O1, O1^i^, O2 and O2^ii^ of the sulfate anion are bonded at the axial positions of Cu1 and Cu2, respectively, resulting in considerably longer Cu—O bonds of 2.393 (2) Å for Cu1—O1 and 2.443 (2) Å for Cu2—O2.

The bridging sulfate ions connect the Cu(4-methylpyridine)_4_ units to form infinite [Cu{H_3_C(C_5_H_4_N)}_4_SO_4_]_n_ zigzag chains along the [001] direction of the crystal (Fig. 2). The shape of the zigzag chain follows the coordination geometry of the copper and sulfate ions: Cu—O—S angles are close to 180° (169.62 (13) for S1—O1—Cu1 and 172.99 (1) for S1—O2—Cu2), the O—Cu—O angles are exactly 180° (due to the location of the copper ions on inversion centers). The angles of the zigzag chain, represented by the Cu—S—Cu angles, thus follow closely the tetrahedral sulfate O—S—O angles and are 111.73 ° (the O—S—O angles range between 108.76 (12) and 110.04 (13)°).

Parallel zigzag chains interdigitate as shown in Fig. 3, but interstitial space is left between neighboring molecules. Along the *a*-axis neighboring sulfate ions are connected through O—H···O hydrogen bonds mediated by the water molecules O5 and O7 to form infinite {O···H_2_O···H_2_O···O···H_2_O···H_2_O···}_n_ chains. Parallel pairs of these chains are connected with each other through additional O—H···O hydrogen bonds mediated by the water molecules of O6 and O8. Due to hydrogen bonding between symmetry equivalent water molecules across inversion centers, the H atoms of O6 and O8 are partially disordered over mutually exclusive positions (see refinement section for details). The connection of the sulfate ions with these water molecules creates flat strands made up of slightly wobbly single molecule layers of tightly hydrogen-bonded water and sulfate molecules about seven to eight Å wide that stretch infinitely parallel to the *a*-axis (Figs. 3 and 4). The last of the five crystallographically independent water solvate molecules (O9) is not part of these strands but is located about three Å away and is only weakly hydrogen bonded with the other water molecules (Fig. 4). It is only partially occupied with a refined occupancy factor of 0.396 (4). For details and numerical values of the hydrogen bonding geometries, including symmetry operators, see: Table 1.

### Experimental

CuSO_4_^.^5H_2_O (0.30 g, 1.21 mmol) was added directly to a stirred solution of pyrolidine (0.5 g, 2.43 mmole) in 20 ml acetone. The contents were stirred until complete dissolution of the salt to which about 30 ml of 4-methylpyridine was added and stirring was continued for another hour. Insoluble matter was removed by filtration and slow evaporation of the reaction mixture at room temperature gave the title compound as blue crystals after three weeks. Yield 60% (0.44 g), m.p. 373 K. Elemental analysis: calculated (found): C 47.15(47.66), H 6.06(5.92), N 9.20(8.95)%.

### Refinement

The crystal under investigation was found to be non-merohedrally twinned by a 180° rotation around the reciprocal *b*-axis. The orientation matrices for the two components were identified using the program *CELL NOW* (Bruker, 2008), and the two components were integrated using *SAINT*, resulting in a total of 23387 reflections. 6633 reflections (4611 unique ones) involved component 1 only (mean I/σ = 12.2), 6573 reflections (3653 unique ones) involved component 2 only (mean I/σ = 5.8), and 10181 reflections (5801 unique ones) involved both components (mean I/σ = 9.4). The exact twin matrix identified by the integration program was found to be -1.00018 0.00039 0.00025, -0.83074 0.99991 - 0.32879, 0.00016 - 0.00153 - 0.99973. The structure was solved using direct methods with only the non-overlapping reflections of component 1. The structure was refined using the HKLF5 routine with all reflections of component 1 (including the overlapping ones) below a *d*-spacing threshold of 0.75 Å, resulting in a BASF value of 0.211 (1). The *R*_int_ value given is for all reflections before the cutoff at d = 0.75 Å and is based on agreement between observed single and composite intensities and those calculated from refined unique intensities and twin fractions (*TWINABS*; Bruker, 2008).

Hydrogen atoms of the water molecules are partially disordered over mutually exclusive positions due to hydrogen bonding between symmetry equivalent water molecules across inversion centers. The H atoms in question are H6A and H8C, which are each located close to a crystallographic inversion center between pairs of symmetry equivalent atoms of O6 and O8. Both H atoms were thus refined as 50% occupied. For both water molecules O6 and O8 a second half occupied hydrogen atom is located in a position in which it hydrogen-bonds with the a neighboring water molecule of O8 and O6, respectively, thus again creating a pair of close by half occupied H atoms (H6C and H8B) in mutually exclusive positions. The water solvate molecule of O9 is only partially occupied with a refined occupancy factor of 0.396 (4). Water hydrogen atoms were located in difference density Fourier maps, assigned occupancies as described above and their positions were refined with an O—H distance of 0.84 (1) Å and H···H distances of 1.30 (1) Å. In the final refinement cycles the water H atoms were set to ride on their carrying oxygen atoms. All other hydrogen atoms were immediately placed in calculated positions and all H atoms were refined with an isotropic displacement parameter *U*_iso_ of 1.5 (methyl, hydroxyl) or 1.2 times (aromatic) that of *U*_eq_ of the adjacent carbon or oxygen atom.

### Crystal data

**Table:**

[Cu(SO_4_)(C_6_H_7_N)_4_]·4.393H_2_O	*Z* = 2
*M**_r_* = 611.27	*F*(000) = 642
Triclinic, *P*1	*D*_x_ = 1.434 Mg m^−^^3^
Hall symbol: -P 1	Melting point: 373 K
*a* = 10.4688 (12) Å	Mo *K*α radiation, λ = 0.71073 Å
*b* = 11.6327 (14) Å	Cell parameters from 4676 reflections
*c* = 12.8300 (15) Å	θ = 2.3–30.4°
α = 78.672 (3)°	µ = 0.90 mm^−^^1^
β = 87.609 (3)°	*T* = 100 K
γ = 67.571 (3)°	Block, blue
*V* = 1415.2 (3) Å^3^	0.60 × 0.45 × 0.40 mm

### Data collection

**Table:**

Bruker SMART APEX CCD diffractometer	6963 independent reflections
Radiation source: fine-focus sealed tube	6170 reflections with *I* > 2σ(*I*)
graphite	*R*_int_ = 0.033
ω scans	θ_max_ = 28.3°, θ_min_ = 1.6°
Absorption correction: multi-scan (*TWINABS*; Bruker, 2008)	*h* = −13→13
*T*_min_ = 0.607, *T*_max_ = 0.746	*k* = −15→15
16814 measured reflections	*l* = 0→17

### Refinement

**Table:**

Refinement on *F*^2^	Primary atom site location: structure-invariant direct methods
Least-squares matrix: full	Secondary atom site location: difference Fourier map
*R*[*F*^2^ > 2σ(*F*^2^)] = 0.046	Hydrogen site location: inferred from neighbouring sites
*wR*(*F*^2^) = 0.118	H-atom parameters constrained
*S* = 1.06	*w* = 1/[σ^2^(*F*_o_^2^) + (0.0573*P*)^2^ + 1.4896*P*] where *P* = (*F*_o_^2^ + 2*F*_c_^2^)/3
6963 reflections	(Δ/σ)_max_ < 0.001
361 parameters	Δρ_max_ = 0.68 e Å^−^^3^
0 restraints	Δρ_min_ = −0.61 e Å^−^^3^

### Special details

**Table:**

Geometry. All e.s.d.'s (except the e.s.d. in the dihedral angle between two l.s. planes) are estimated using the full covariance matrix. The cell e.s.d.'s are taken into account individually in the estimation of e.s.d.'s in distances, angles and torsion angles; correlations between e.s.d.'s in cell parameters are only used when they are defined by crystal symmetry. An approximate (isotropic) treatment of cell e.s.d.'s is used for estimating e.s.d.'s involving l.s. planes.
Refinement. Refinement of *F*^2^ against ALL reflections. The weighted *R*-factor *wR* and goodness of fit *S* are based on *F*^2^, conventional *R*-factors *R* are based on *F*, with *F* set to zero for negative *F*^2^. The threshold expression of *F*^2^ > σ(*F*^2^) is used only for calculating *R*-factors(gt) *etc*. and is not relevant to the choice of reflections for refinement. *R*-factors based on *F*^2^ are statistically about twice as large as those based on *F*, and *R*- factors based on ALL data will be even larger.

### Fractional atomic coordinates and isotropic or equivalent isotropic displacement parameters (Å^2^)

**Table:**

	*x*	*y*	*z*	*U*_iso_*/*U*_eq_	Occ. (<1)
C1	0.7599 (3)	1.0000 (3)	0.5809 (2)	0.0204 (5)	
H1	0.7594	0.9263	0.6281	0.024*	
C2	0.8641 (3)	1.0426 (3)	0.5935 (2)	0.0231 (5)	
H2	0.9329	0.9984	0.6488	0.028*	
C3	0.8680 (3)	1.1497 (3)	0.5255 (2)	0.0230 (5)	
C4	0.7680 (3)	1.2070 (3)	0.4436 (2)	0.0254 (6)	
H4	0.7692	1.2784	0.3932	0.030*	
C5	0.6665 (3)	1.1597 (3)	0.4355 (2)	0.0242 (6)	
H5	0.5990	1.1999	0.3789	0.029*	
C6	0.9755 (3)	1.2030 (3)	0.5399 (3)	0.0313 (6)	
H6F	0.9338	1.2775	0.5729	0.047*	
H6E	1.0100	1.2278	0.4704	0.047*	
H6D	1.0523	1.1385	0.5856	0.047*	
C7	0.6771 (3)	0.7248 (3)	0.5052 (2)	0.0259 (6)	
H7	0.6453	0.7125	0.5754	0.031*	
C8	0.7656 (4)	0.6209 (3)	0.4678 (2)	0.0318 (7)	
H8	0.7943	0.5392	0.5123	0.038*	
C9	0.8136 (3)	0.6346 (3)	0.3647 (2)	0.0272 (6)	
C10	0.9073 (4)	0.5230 (3)	0.3195 (3)	0.0451 (9)	
H10A	0.8548	0.4736	0.3050	0.068*	
H10B	0.9834	0.4695	0.3709	0.068*	
H10C	0.9449	0.5532	0.2533	0.068*	
C11	0.7700 (3)	0.7568 (3)	0.3053 (2)	0.0224 (5)	
H11	0.8015	0.7715	0.2353	0.027*	
C12	0.6808 (3)	0.8575 (3)	0.3476 (2)	0.0212 (5)	
H12	0.6517	0.9402	0.3048	0.025*	
C13	0.4334 (3)	0.8651 (3)	0.2020 (2)	0.0216 (5)	
H13	0.3870	0.9490	0.2138	0.026*	
C14	0.4296 (3)	0.7646 (3)	0.2792 (2)	0.0208 (5)	
H14	0.3811	0.7806	0.3424	0.025*	
C15	0.4963 (3)	0.6413 (3)	0.2645 (2)	0.0253 (6)	
C16	0.5674 (4)	0.6247 (3)	0.1714 (2)	0.0341 (7)	
H16	0.6166	0.5416	0.1585	0.041*	
C17	0.5667 (3)	0.7287 (3)	0.0975 (2)	0.0310 (6)	
H17	0.6156	0.7150	0.0342	0.037*	
C18	0.4934 (4)	0.5303 (3)	0.3466 (2)	0.0371 (8)	
H18A	0.5735	0.5000	0.3960	0.056*	
H18B	0.4967	0.4620	0.3112	0.056*	
H18C	0.4082	0.5569	0.3860	0.056*	
C19	0.7394 (3)	1.0233 (3)	0.0928 (2)	0.0228 (5)	
H19	0.6692	1.0967	0.1105	0.027*	
C20	0.8748 (3)	0.9916 (3)	0.1272 (2)	0.0258 (6)	
H20	0.8958	1.0428	0.1676	0.031*	
C21	0.9792 (3)	0.8852 (3)	0.1023 (2)	0.0256 (6)	
C22	0.9428 (3)	0.8158 (3)	0.0406 (2)	0.0291 (6)	
H22	1.0113	0.7430	0.0207	0.035*	
C23	0.8054 (3)	0.8539 (3)	0.0084 (2)	0.0272 (6)	
H23	0.7822	0.8067	−0.0349	0.033*	
C24	1.1264 (3)	0.8448 (3)	0.1420 (2)	0.0328 (7)	
H24A	1.1478	0.9201	0.1405	0.049*	
H24B	1.1893	0.7918	0.0962	0.049*	
H24C	1.1378	0.7964	0.2150	0.049*	
Cu1	0.5000	1.0000	0.5000	0.01857 (11)	
Cu2	0.5000	1.0000	0.0000	0.02031 (11)	
N1	0.6596 (2)	1.0589 (2)	0.50462 (17)	0.0197 (4)	
N2	0.6332 (2)	0.8438 (2)	0.44667 (17)	0.0196 (4)	
N3	0.5002 (2)	0.8479 (2)	0.11114 (17)	0.0204 (4)	
N4	0.7039 (2)	0.9544 (2)	0.03565 (17)	0.0208 (4)	
O1	0.4432 (2)	1.11465 (19)	0.32076 (14)	0.0241 (4)	
O2	0.4297 (2)	1.1372 (2)	0.13099 (15)	0.0257 (4)	
O3	0.2438 (2)	1.2743 (2)	0.21897 (18)	0.0387 (6)	
O4	0.4641 (3)	1.2969 (2)	0.20710 (19)	0.0389 (6)	
O5	0.7247 (3)	0.3043 (2)	0.1438 (2)	0.0501 (7)	
H5A	0.7007	0.3519	0.0826	0.075*	
H5B	0.6529	0.2956	0.1714	0.075*	
O6	0.3674 (3)	0.5172 (3)	0.0548 (2)	0.0568 (7)	
H6A	0.4103	0.5011	−0.0008	0.085*	0.50
H6B	0.4034	0.4467	0.0972	0.085*	
H6C	0.2905	0.5147	0.0399	0.085*	0.50
O7	0.9960 (3)	0.2146 (3)	0.2479 (2)	0.0567 (8)	
H7A	0.9293	0.2632	0.2054	0.085*	
H7B	1.0552	0.2449	0.2228	0.085*	
O8	0.1306 (4)	0.4630 (4)	0.0397 (4)	0.0971 (15)	
H8A	0.1109	0.4722	0.1027	0.146*	
H8B	0.1842	0.5027	0.0261	0.146*	0.50
H8C	0.0507	0.4844	0.0125	0.146*	0.50
O9	0.2000 (7)	0.5327 (6)	0.2704 (5)	0.045 (2)	0.393 (8)
H9A	0.2311	0.5640	0.2151	0.067*	0.393 (8)
H9B	0.2166	0.4593	0.2590	0.067*	0.393 (8)
S3	0.39467 (7)	1.20521 (6)	0.21937 (5)	0.01904 (13)	

### Atomic displacement parameters (Å^2^)

**Table:**

	*U*^11^	*U*^22^	*U*^33^	*U*^12^	*U*^13^	*U*^23^
C1	0.0229 (13)	0.0205 (12)	0.0186 (12)	−0.0087 (11)	−0.0004 (9)	−0.0046 (10)
C2	0.0201 (12)	0.0294 (14)	0.0220 (13)	−0.0095 (11)	−0.0012 (10)	−0.0099 (11)
C3	0.0187 (12)	0.0275 (14)	0.0274 (14)	−0.0106 (11)	0.0047 (10)	−0.0131 (11)
C4	0.0276 (14)	0.0269 (14)	0.0247 (14)	−0.0145 (12)	0.0019 (11)	−0.0035 (11)
C5	0.0278 (14)	0.0285 (14)	0.0198 (13)	−0.0162 (12)	−0.0028 (10)	−0.0002 (10)
C6	0.0259 (14)	0.0341 (16)	0.0411 (17)	−0.0166 (13)	0.0008 (12)	−0.0126 (13)
C7	0.0371 (16)	0.0245 (14)	0.0176 (12)	−0.0145 (12)	−0.0017 (11)	−0.0015 (10)
C8	0.0478 (19)	0.0231 (14)	0.0235 (14)	−0.0138 (14)	−0.0010 (13)	−0.0013 (11)
C9	0.0316 (15)	0.0267 (15)	0.0241 (14)	−0.0111 (12)	−0.0026 (11)	−0.0056 (11)
C10	0.062 (2)	0.0287 (17)	0.0363 (19)	−0.0067 (17)	0.0047 (17)	−0.0091 (14)
C11	0.0238 (13)	0.0278 (14)	0.0184 (12)	−0.0128 (11)	0.0005 (10)	−0.0046 (10)
C12	0.0202 (12)	0.0258 (13)	0.0190 (12)	−0.0118 (11)	−0.0009 (10)	−0.0014 (10)
C13	0.0212 (13)	0.0235 (13)	0.0222 (13)	−0.0106 (11)	−0.0007 (10)	−0.0044 (10)
C14	0.0221 (12)	0.0275 (14)	0.0178 (12)	−0.0145 (11)	0.0002 (10)	−0.0051 (10)
C15	0.0333 (15)	0.0262 (14)	0.0187 (13)	−0.0148 (12)	−0.0032 (11)	−0.0017 (10)
C16	0.053 (2)	0.0220 (14)	0.0234 (14)	−0.0103 (14)	0.0054 (13)	−0.0057 (11)
C17	0.0429 (18)	0.0297 (15)	0.0183 (13)	−0.0117 (14)	0.0062 (12)	−0.0053 (11)
C18	0.062 (2)	0.0293 (16)	0.0227 (15)	−0.0231 (16)	0.0065 (14)	−0.0013 (12)
C19	0.0259 (13)	0.0262 (14)	0.0201 (12)	−0.0157 (11)	−0.0012 (10)	−0.0010 (10)
C20	0.0290 (14)	0.0341 (15)	0.0208 (13)	−0.0211 (13)	−0.0035 (10)	−0.0006 (11)
C21	0.0212 (13)	0.0377 (16)	0.0175 (12)	−0.0147 (12)	−0.0016 (10)	0.0032 (11)
C22	0.0232 (14)	0.0358 (16)	0.0252 (14)	−0.0078 (12)	−0.0004 (11)	−0.0059 (12)
C23	0.0280 (14)	0.0338 (15)	0.0223 (13)	−0.0134 (13)	−0.0044 (11)	−0.0065 (12)
C24	0.0235 (14)	0.0469 (18)	0.0278 (15)	−0.0162 (14)	−0.0024 (11)	−0.0009 (13)
Cu1	0.0212 (2)	0.0220 (2)	0.0169 (2)	−0.01266 (19)	−0.00015 (16)	−0.00454 (18)
Cu2	0.0198 (2)	0.0240 (2)	0.0176 (2)	−0.0110 (2)	−0.00355 (16)	0.00135 (18)
N1	0.0229 (11)	0.0248 (11)	0.0158 (10)	−0.0127 (9)	0.0005 (8)	−0.0060 (8)
N2	0.0237 (11)	0.0233 (11)	0.0163 (10)	−0.0141 (9)	−0.0028 (8)	−0.0025 (8)
N3	0.0213 (11)	0.0236 (11)	0.0178 (10)	−0.0103 (9)	−0.0041 (8)	−0.0027 (9)
N4	0.0221 (11)	0.0242 (11)	0.0172 (10)	−0.0119 (9)	−0.0015 (8)	0.0003 (8)
O1	0.0311 (10)	0.0295 (10)	0.0130 (8)	−0.0145 (9)	−0.0030 (7)	−0.0002 (7)
O2	0.0331 (11)	0.0319 (11)	0.0149 (9)	−0.0143 (9)	0.0015 (8)	−0.0069 (8)
O3	0.0250 (11)	0.0461 (14)	0.0315 (12)	−0.0004 (10)	0.0011 (9)	−0.0046 (10)
O4	0.0571 (15)	0.0289 (11)	0.0373 (13)	−0.0282 (11)	−0.0223 (11)	0.0077 (9)
O5	0.0516 (16)	0.0386 (14)	0.0540 (16)	−0.0131 (12)	−0.0121 (13)	−0.0011 (12)
O6	0.077 (2)	0.0500 (17)	0.0409 (15)	−0.0260 (16)	−0.0023 (14)	0.0007 (13)
O7	0.0577 (17)	0.0562 (17)	0.0492 (16)	−0.0260 (15)	−0.0123 (13)	0.0174 (14)
O8	0.066 (2)	0.089 (3)	0.123 (3)	−0.044 (2)	−0.044 (2)	0.050 (2)
O9	0.049 (4)	0.044 (4)	0.045 (4)	−0.017 (3)	0.001 (3)	−0.017 (3)
S3	0.0231 (3)	0.0207 (3)	0.0147 (3)	−0.0099 (2)	−0.0032 (2)	−0.0026 (2)

### Geometric parameters (Å, °)

**Table:**

C1—N1	1.343 (3)	C19—N4	1.342 (3)
C1—C2	1.386 (4)	C19—C20	1.388 (4)
C1—H1	0.9500	C19—H19	0.9500
C2—C3	1.387 (4)	C20—C21	1.386 (4)
C2—H2	0.9500	C20—H20	0.9500
C3—C4	1.389 (4)	C21—C22	1.391 (4)
C3—C6	1.509 (4)	C21—C24	1.508 (4)
C4—C5	1.386 (4)	C22—C23	1.388 (4)
C4—H4	0.9500	C22—H22	0.9500
C5—N1	1.348 (4)	C23—N4	1.341 (4)
C5—H5	0.9500	C23—H23	0.9500
C6—H6F	0.9800	C24—H24A	0.9800
C6—H6E	0.9800	C24—H24B	0.9800
C6—H6D	0.9800	C24—H24C	0.9800
C7—N2	1.352 (4)	Cu1—N1^i^	2.041 (2)
C7—C8	1.373 (4)	Cu1—N1	2.041 (2)
C7—H7	0.9500	Cu1—N2	2.046 (2)
C8—C9	1.397 (4)	Cu1—N2^i^	2.046 (2)
C8—H8	0.9500	Cu1—O1	2.3926 (18)
C9—C11	1.385 (4)	Cu1—O1^i^	2.3926 (18)
C9—C10	1.503 (4)	Cu2—N4^ii^	2.042 (2)
C10—H10A	0.9800	Cu2—N4	2.042 (2)
C10—H10B	0.9800	Cu2—N3	2.042 (2)
C10—H10C	0.9800	Cu2—N3^ii^	2.042 (2)
C11—C12	1.382 (4)	Cu2—O2^ii^	2.443 (2)
C11—H11	0.9500	Cu2—O2	2.443 (2)
C12—N2	1.348 (3)	O1—S3	1.4726 (19)
C12—H12	0.9500	O2—S3	1.464 (2)
C13—N3	1.344 (3)	O3—S3	1.473 (2)
C13—C14	1.387 (4)	O4—S3	1.485 (2)
C13—H13	0.9500	O5—H5A	0.8515
C14—C15	1.382 (4)	O5—H5B	0.8498
C14—H14	0.9500	O6—H6A	0.8408
C15—C16	1.388 (4)	O6—H6B	0.8470
C15—C18	1.507 (4)	O6—H6C	0.8472
C16—C17	1.381 (4)	O7—H7A	0.8445
C16—H16	0.9500	O7—H7B	0.8477
C17—N3	1.336 (4)	O8—H8A	0.8431
C17—H17	0.9500	O8—H8B	0.8459
C18—H18A	0.9800	O8—H8C	0.8456
C18—H18B	0.9800	O9—H9A	0.8436
C18—H18C	0.9800	O9—H9B	0.8448
			
N1—C1—C2	122.6 (3)	C22—C21—C24	121.1 (3)
N1—C1—H1	118.7	C23—C22—C21	119.3 (3)
C2—C1—H1	118.7	C23—C22—H22	120.3
C1—C2—C3	120.0 (3)	C21—C22—H22	120.3
C1—C2—H2	120.0	N4—C23—C22	123.0 (3)
C3—C2—H2	120.0	N4—C23—H23	118.5
C2—C3—C4	117.3 (2)	C22—C23—H23	118.5
C2—C3—C6	121.7 (3)	C21—C24—H24A	109.5
C4—C3—C6	121.0 (3)	C21—C24—H24B	109.5
C5—C4—C3	119.7 (3)	H24A—C24—H24B	109.5
C5—C4—H4	120.1	C21—C24—H24C	109.5
C3—C4—H4	120.1	H24A—C24—H24C	109.5
N1—C5—C4	122.7 (3)	H24B—C24—H24C	109.5
N1—C5—H5	118.7	N1^i^—Cu1—N1	180.00 (11)
C4—C5—H5	118.7	N1^i^—Cu1—N2	91.28 (9)
C3—C6—H6F	109.5	N1—Cu1—N2	88.72 (9)
C3—C6—H6E	109.5	N1^i^—Cu1—N2^i^	88.72 (9)
H6F—C6—H6E	109.5	N1—Cu1—N2^i^	91.28 (9)
C3—C6—H6D	109.5	N2—Cu1—N2^i^	179.999 (1)
H6F—C6—H6D	109.5	N1^i^—Cu1—O1	90.27 (8)
H6E—C6—H6D	109.5	N1—Cu1—O1	89.73 (8)
N2—C7—C8	122.9 (3)	N2—Cu1—O1	90.38 (8)
N2—C7—H7	118.5	N2^i^—Cu1—O1	89.62 (8)
C8—C7—H7	118.5	N1^i^—Cu1—O1^i^	89.73 (8)
C7—C8—C9	120.4 (3)	N1—Cu1—O1^i^	90.27 (8)
C7—C8—H8	119.8	N2—Cu1—O1^i^	89.62 (8)
C9—C8—H8	119.8	N2^i^—Cu1—O1^i^	90.38 (8)
C11—C9—C8	116.6 (3)	O1—Cu1—O1^i^	180.0
C11—C9—C10	121.3 (3)	N4^ii^—Cu2—N4	180.0
C8—C9—C10	122.1 (3)	N4^ii^—Cu2—N3	89.63 (9)
C9—C10—H10A	109.5	N4—Cu2—N3	90.37 (9)
C9—C10—H10B	109.5	N4^ii^—Cu2—N3^ii^	90.37 (9)
H10A—C10—H10B	109.5	N4—Cu2—N3^ii^	89.63 (9)
C9—C10—H10C	109.5	N3—Cu2—N3^ii^	179.999 (1)
H10A—C10—H10C	109.5	N4—Cu2—O2^ii^	88.90 (8)
H10B—C10—H10C	109.5	N4^ii^—Cu2—O2^ii^	91.11 (8)
C12—C11—C9	120.2 (3)	N3—Cu2—O2^ii^	88.72 (8)
C12—C11—H11	119.9	N3^ii^—Cu2—O2^ii^	91.28 (8)
C9—C11—H11	119.9	N4—Cu2—O2	91.11 (8)
N2—C12—C11	123.1 (3)	N4^ii^—Cu2—O2	88.90 (8)
N2—C12—H12	118.5	N3—Cu2—O2	91.28 (8)
C11—C12—H12	118.5	N3^ii^—Cu2—O2	88.72 (8)
N3—C13—C14	122.4 (3)	O2^ii^—Cu2—O2	180.000 (1)
N3—C13—H13	118.8	C1—N1—C5	117.5 (2)
C14—C13—H13	118.8	C1—N1—Cu1	120.11 (18)
C15—C14—C13	120.2 (3)	C5—N1—Cu1	122.30 (18)
C15—C14—H14	119.9	C12—N2—C7	116.8 (2)
C13—C14—H14	119.9	C12—N2—Cu1	119.50 (19)
C14—C15—C16	116.9 (3)	C7—N2—Cu1	123.72 (19)
C14—C15—C18	121.5 (3)	C17—N3—C13	117.5 (2)
C16—C15—C18	121.7 (3)	C17—N3—Cu2	122.05 (19)
C17—C16—C15	120.1 (3)	C13—N3—Cu2	120.41 (19)
C17—C16—H16	120.0	C23—N4—C19	117.7 (2)
C15—C16—H16	120.0	C23—N4—Cu2	122.75 (19)
N3—C17—C16	122.9 (3)	C19—N4—Cu2	119.52 (19)
N3—C17—H17	118.5	S3—O1—Cu1	169.60 (13)
C16—C17—H17	118.5	H5A—O5—H5B	107.6
C15—C18—H18A	109.5	H6A—O6—H6B	101.2
C15—C18—H18B	109.5	H6A—O6—H6C	101.0
H18A—C18—H18B	109.5	H6B—O6—H6C	100.4
C15—C18—H18C	109.5	H7A—O7—H7B	97.7
H18A—C18—H18C	109.5	H8A—O8—H8B	100.6
H18B—C18—H18C	109.5	H8A—O8—H8C	100.5
N4—C19—C20	122.5 (3)	H8B—O8—H8C	126.9
N4—C19—H19	118.7	H9A—O9—H9B	100.6
C20—C19—H19	118.7	O2—S3—O1	109.72 (12)
C21—C20—C19	119.9 (3)	O2—S3—O3	109.57 (13)
C21—C20—H20	120.0	O1—S3—O3	110.06 (13)
C19—C20—H20	120.0	O2—S3—O4	109.34 (14)
C20—C21—C22	117.5 (3)	O1—S3—O4	108.76 (12)
C20—C21—C24	121.4 (3)	O3—S3—O4	109.37 (15)
			
N1—C1—C2—C3	−0.3 (4)	C11—C12—N2—C7	0.1 (4)
C1—C2—C3—C4	−2.6 (4)	C11—C12—N2—Cu1	−179.4 (2)
C1—C2—C3—C6	176.9 (3)	C8—C7—N2—C12	−0.1 (4)
C2—C3—C4—C5	2.7 (4)	C8—C7—N2—Cu1	179.4 (2)
C6—C3—C4—C5	−176.8 (3)	N1^i^—Cu1—N2—C12	109.81 (19)
C3—C4—C5—N1	0.1 (5)	N1—Cu1—N2—C12	−70.19 (19)
N2—C7—C8—C9	−0.7 (5)	O1—Cu1—N2—C12	19.53 (19)
C7—C8—C9—C11	1.5 (4)	O1^i^—Cu1—N2—C12	−160.47 (19)
C7—C8—C9—C10	−178.2 (3)	N1^i^—Cu1—N2—C7	−69.7 (2)
C8—C9—C11—C12	−1.5 (4)	N1—Cu1—N2—C7	110.3 (2)
C10—C9—C11—C12	178.3 (3)	O1—Cu1—N2—C7	−160.0 (2)
C9—C11—C12—N2	0.7 (4)	O1^i^—Cu1—N2—C7	20.0 (2)
N3—C13—C14—C15	0.1 (4)	C16—C17—N3—C13	1.0 (5)
C13—C14—C15—C16	1.2 (4)	C16—C17—N3—Cu2	−179.6 (3)
C13—C14—C15—C18	−179.7 (3)	C14—C13—N3—C17	−1.2 (4)
C14—C15—C16—C17	−1.4 (5)	C14—C13—N3—Cu2	179.34 (19)
C18—C15—C16—C17	179.4 (3)	N4^ii^—Cu2—N3—C17	109.5 (2)
C15—C16—C17—N3	0.4 (5)	N4—Cu2—N3—C17	−70.5 (2)
N4—C19—C20—C21	0.1 (4)	N4^ii^—Cu2—N3—C13	−71.1 (2)
C19—C20—C21—C22	1.6 (4)	N4—Cu2—N3—C13	108.9 (2)
C19—C20—C21—C24	−177.5 (3)	C22—C23—N4—C19	2.9 (4)
C20—C21—C22—C23	−1.1 (4)	C22—C23—N4—Cu2	−174.0 (2)
C24—C21—C22—C23	178.1 (3)	C20—C19—N4—C23	−2.3 (4)
C21—C22—C23—N4	−1.2 (5)	C20—C19—N4—Cu2	174.7 (2)
C2—C1—N1—C5	3.2 (4)	N3—Cu2—N4—C23	71.8 (2)
C2—C1—N1—Cu1	−174.3 (2)	N3^ii^—Cu2—N4—C23	−108.2 (2)
C4—C5—N1—C1	−3.1 (4)	N3—Cu2—N4—C19	−105.1 (2)
C4—C5—N1—Cu1	174.3 (2)	N3^ii^—Cu2—N4—C19	74.9 (2)
N2—Cu1—N1—C1	−73.2 (2)	N1^i^—Cu1—O1—S3	97.0 (7)
N2^i^—Cu1—N1—C1	106.8 (2)	N1—Cu1—O1—S3	−83.0 (7)
O1—Cu1—N1—C1	−163.6 (2)	N2—Cu1—O1—S3	−171.7 (7)
O1^i^—Cu1—N1—C1	16.4 (2)	N2^i^—Cu1—O1—S3	8.3 (7)
N2—Cu1—N1—C5	109.4 (2)	Cu1—O1—S3—O2	−173.0 (7)
N2^i^—Cu1—N1—C5	−70.6 (2)	Cu1—O1—S3—O3	−52.3 (7)
O1—Cu1—N1—C5	19.1 (2)	Cu1—O1—S3—O4	67.4 (7)
O1^i^—Cu1—N1—C5	−160.9 (2)		

Symmetry codes: (i) −*x*+1, −*y*+2, −*z*+1; (ii) −*x*+1, −*y*+2, −*z*.

### Hydrogen-bond geometry (Å, °)

**Table:**

*D*—H···*A*	*D*—H	H···*A*	*D*···*A*	*D*—H···*A*
O5—H5A···O6^iii^	0.85	2.04	2.887 (4)	173
O5—H5B···O4^iv^	0.85	2.01	2.843 (4)	168
O6—H6A···O6^iii^	0.84	2.34	2.983 (6)	133
O6—H6B···O4^iv^	0.85	1.92	2.762 (4)	172
O6—H6C···O8	0.85	1.98	2.804 (5)	163
O7—H7A···O5	0.84	2.16	2.906 (4)	148
O7—H7B···O3^v^	0.85	2.13	2.923 (4)	157
O8—H8A···O3^iv^	0.84	2.42	2.788 (4)	107
O8—H8B···O6	0.85	2.04	2.804 (5)	149
O8—H8C···O8^vi^	0.85	1.87	2.710 (7)	176
O9—H9A···O6	0.84	2.48	3.210 (7)	145
O9—H9B···O3^iv^	0.84	2.22	3.063 (7)	175
O9—H9B···O4^iv^	0.84	2.71	3.275 (7)	126

Symmetry codes: (iii) −*x*+1, −*y*+1, −*z*; (iv) *x*, *y*−1, *z*; (v) *x*+1, *y*−1, *z*; (vi) −*x*, −*y*+1, −*z*.
